# 自动上样固相萃取-超高效液相色谱-串联质谱法同时测定水中9类43种抗菌药物残留

**DOI:** 10.3724/SP.J.1123.2022.09008

**Published:** 2023-07-08

**Authors:** Baolin XIA, Shitao WANG, Jingjing YIN, Weiyi ZHANG, Na YANG, Qiang LIU, Haijing WU

**Affiliations:** 1.江阴市食品安全检测中心,江苏江阴214400; 1. Testing Center of Jiangyin Food Safety, Jiangyin 214400, China; 2.南京财经大学食品科学与工程学院, 江苏南京210023; 2. College of Food Science and Engineering, Nanjing University of Finance and Economics, Nanjing 210023, China; 3.南京市食品药品监督检验院,江苏南京211198; 3. Nanjing Institute for Food and Drug Control, Nanjing 211198, China

**Keywords:** 超高效液相色谱-串联质谱, 固相萃取, 自动上样, 抗菌药物残留, 水体, ultra performance liquid chromatography-tandem mass spectrometry (UPLC-MS/MS), solid phase extraction (SPE), automatic sample loading, antibacterial residue, water body

## Abstract

为了实现水中抗菌药物快速、准确、高通量的分析,采用自动上样固相萃取-超高效液相色谱-串联质谱技术,建立了一种能够快速、稳定地分析水中9类(磺胺类、喹诺酮类、氟喹诺酮类、四环素类、林可酰胺类、大环内酯类、硝基咪唑类、双萜烯类、二氢叶酸还原酶抑制剂类)43种抗菌药物多残留同步分析方法。水样经0.45 μm水相滤膜过滤,加入一定量的磷酸二氢钠及乙二胺四乙酸二钠,并用磷酸调节pH至2.34,加入内标混匀,使用自制的自动上样装置进行上样,Oasis HLB固相萃取柱富集净化。利用Waters Acquity UPLC BEH C_18_柱(50 mm×2.1 mm, 1.7 μm)进行分离,以含0.1%甲酸的甲醇-乙腈(2∶8, v/v)混合溶液-0.1%甲酸水溶液为流动相进行梯度洗脱,采用电喷雾正离子多反应监测模式进行分段扫描,内、外标法相结合的方式分析目标物。结果显示,43种抗菌药物在各自的线性范围内线性关系良好,方法的检出限为0.004~1.000 ng/L,定量限为0.012~3.000 ng/L,加标回收率为53.7%~130.4%,相对标准偏差为0.9%~13.2%。采用该方法对取自长江江阴段、锡澄运河江阴段各3份水样以及6份自来水样进行检测。6份自来水中均未检测到抗菌药物,6份取自长江江阴段、锡澄运河江阴段的水样中共检出20种抗菌药物,除四环素类外,其余类别均有检出,其中,以磺胺甲恶唑含量最高(8.92~11.03 ng/L),泰妙菌素和沃尼妙林两种双萜烯类抗菌药物在自然水体中普遍有检出。该法准确、灵敏、快速,适用于水中43种抗菌药物的检测。

## Abstract (en)

Antibacterials represent a pharmaceutical class that is extensively used and consumed worldwide. The presence of a large number of antibacterial agents in water could result in antibiotic resistance. Thus, the development of a fast, accurate, and high-throughput method to analyze these emerging contaminants in water is necessary. Herein, a method was developed to achieve the simultaneous determination of 43 antibacterials from nine pharmaceutical categories (i.e., sulfonamides, quinolones, fluoroquinolones, tetracyclines, lincosamides, macrolides, nitroimidazoles, diterpenes, and dihydrofolate reductase inhibitors) in water using automatic sample loading-solid phase extraction (SPE)-ultra performance liquid chromatography-tandem mass spectrometry (UPLC-MS/MS). Because the properties of these 43 antibacterials are quite different, the main objective of this work is to develop an extraction procedure that would enable the simultaneous analysis of a wide range of multiclass antibacterials. Given this context, the work presented in this paper optimized the SPE cartridge type, pH, and sample loading amount.

Multiresidue extraction was performed as follows. The water samples were filtered through 0.45 μm filter membranes, added with Na_2_EDTA and NaH_2_PO_4_, and pH-adjusted to 2.34 using H_3_PO_4_. The solutions were then mixed with the internal standards. An automatic sample loading device fabricated by the authors was used for sample loading, and Oasis HLB cartridges were used for enrichment and purification. The optimized UPLC conditions were as follows: chromatographic column, Waters Acquity UPLC BEH C_18_ column (50 mm×2.1 mm, 1.7 μm); mobile phases, methanol-acetonitrile (2∶8, v/v) solution containing 0.1% formic acid and 0.1% formic acid aqueous solution; flow rate, 0.3 mL/min; injection volume, 10 μL. The compounds were step scanned using an electrospray ionization source in the positive and multiple-reaction monitoring (MRM) modes, and analyzed by internal and external standard methods.

The results showed that the 43 compounds achieved high linearity in their respective linear ranges, with correlation coefficients (*r*^2^) greater than 0.996. The limits of detection (LODs) of the 43 antibacterial agents ranged from 0.004 ng/L to 1.000 ng/L, and their limits of quantification (LOQs) ranged from 0.012 ng/L to 3.000 ng/L. The average recoveries ranged from 53.7% to 130.4%, and the relative standard deviations (RSDs) were between 0.9% and 13.2%. The method was successfully applied to the determination of six tap water samples from different districts and six water samples obtained from the Jiangyin section of the Yangtze River and Xicheng Canal. No antibacterial compound was detected in any of the tap water samples, but a total of 20 antibacterial compounds were detected in the river and canal water samples. Among these compounds, sulfamethoxazole showed the highest mass concentrations, ranging from 8.92 to 11.03 ng/L. The types and contents of antibacterials detected in the Xicheng Canal were greater than those found in the Yangtze River, and two kinds of diterpenes, namely tiamulin and valnemulin, were found easily and commonly in water sample. The findings indicate that antibacterial agents are widespread in environmental waters. The developed method is accurate, sensitive, rapid, and suitable for the detection of the 43 antibacterial compounds in water samples.

抗菌药物是世界范围内使用和消费较高的药物类别之一。抗菌药物被广泛用于治疗细菌感染,同时,在牲畜生产、鱼类养殖、家禽养殖等领域,抗菌药物也作为生长促进剂、饲料添加剂、抗球虫抑制剂被大量使用^[1,2]^。近年来,在环境水域(废水、地表水和饮用水)中不断检测到各种抗菌药物残留,其种类主要包括磺胺类、大环内酯类、四环素类、喹诺酮类、林可酰胺类、酰胺醇类等,暴露水平一般为ng/L~μg/L级别^[3⇓⇓⇓⇓-8]^。抗菌药物在较低水平下即可对病原菌产生作用,导致耐药性的产生。目前,自然环境中已经发现了“超级细菌”,其耐药基因可以通过水和食物链转移到人类相关细菌中,从而促进耐药增殖,这必然对生态环境以及人类健康产生极大的负面影响^[9]^。所以,有必要建立快速、灵敏和可靠的分析方法,以便能够在较低水平上检测水体中低浓度的抗菌药物残留。

高效液相色谱-串联质谱法(HPLC-MS/MS)因具有高灵敏度、高选择性、强抗干扰能力等特点已经广泛用于水体中多种抗菌药物的测定。Gros等^[6]^建立了固相萃取(SPE)-LC-MS/MS对医院污水、城市废水、河水中10类53种抗菌药物及其代谢产物同步测定的分析方法。封梦娟等^[3]^建立了SPE-HPLC-MS/MS同时测定表层水中5类40种抗菌药物的分析方法,该方法高效、灵敏、可靠,适用于实际水样中多种抗菌药物残留量的分析,回收率在41.3%~112.6%之间。谢恺等^[10]^建立了水中4种大环内酯类抗菌药物的固相萃取-超高效液相色谱-串联质谱(SPE-UPLC-MS/MS)的测定方法,该方法简便、快速、灵敏,适用于末梢水中4种痕量大环内酯类的检测。由于水体中抗菌药物含量低,目标物的富集尤为重要。SPE技术是文献中常用的水样富集方式^[3,6⇓-8]^。王蕴馨等^[8]^采用全自动SPE装置实现了生活饮用水及水源水中13种抗生素富集。但该装置价格昂贵、维护成本高、普及率低等,影响全自动固相萃取技术的推广。孙慧婧等^[4]^采用大体积直接进样的方法实现了水中7类42种抗菌药物残留的测定。该方法对仪器灵敏度要求高,且由于进样量达到100 μL,同时乙二胺四乙酸二钠(Na_2_EDTA)为非挥发性盐,仪器污染风险加大。文献[2,3,5,6]中采用手动上样,固相萃取柱富集净化,一般固相萃取柱容量为3 mL或6 mL,由于上样量大(可达1 L),导致前处理过程操作麻烦,费时费力,且过程不可控。

本文设计了一套自动上样装置,该装置与固相萃取装置进行耦合,可实现水样富集净化过程全自动化,同时对固相萃取柱柱型选择、pH值、上样体积等参数进行优化,建立了水中9类43种抗菌药物(12种磺胺类(SAs)、2种喹诺酮类(QNs)、12种氟喹诺酮类(FQs)、4种四环素类(TCs)、1种林可酰胺类(LINs)、5种大环内酯类(MLs)、4种硝基咪唑类(NMs)、2种双萜烯类(DTs)、1种二氢叶酸还原酶抑制剂类(DRIs))的多残留同步分析方法,并应用于生活饮用水、环境水等水体中抗菌药物残留量的测定。

## 1 实验部分

### 1.1 仪器、材料与试剂

Acquity UPLC XEVO TQS超高效液相色谱-串联质谱仪(美国Waters公司);MV5浓缩仪(北京莱伯泰科有限公司); DOA-P504-BN真空泵(美国GAST公司);VISIPREP DL固相萃取装置(美国Supelco公司) 。Oasis HLB固相萃取小柱和Oasis MCX固相萃取小柱(规格均为500 g/6 mL,美国Waters公司);0.45 μm水相滤膜(上海安谱实验科技股份有限公司)。

实验用水为超纯水(美国Thermo Fisher公司);甲醇、乙腈、甲酸、氨水(色谱纯,德国Merck公司); Na_2_EDTA、磷酸二氢钾、磷酸(分析纯,天津科密欧化学试剂有限公司)。

43种抗菌药物标准品:(1)磺胺类:磺胺甲恶唑(SMX)、磺胺二甲基嘧啶(SMZ)、磺胺间甲氧嘧啶(SMM)、磺胺喹噁啉(SQ)、磺胺间二甲氧嘧啶(SDM)、磺胺嘧啶(SDZ)、磺胺噻唑(STZ)、磺胺甲基嘧啶(SMR)、磺胺二甲异恶唑(SIZ)、磺胺甲噻二唑(SMTZ)、磺胺氯哒嗪(SCP)、磺胺邻二甲氧嘧啶(SDX); (2)喹诺酮类:噁喹酸(OXO)、氟甲喹(FLU); (3)氟喹诺酮类:恩诺沙星(ENR)、环丙沙星(CIP)、氧氟沙星(OFL)、诺氟沙星(NOR)、依诺沙星(ENO)、培氟沙星(PEF)、洛美沙星(LOM)、达氟沙星(DAN)、双氟沙星(DIF)、氟罗沙星(FLE)、沙拉沙星(SAR)、斯帕沙星(SPX); (4)四环素类:多西环素(DC)、西环素(TC)、土霉素(OTC)、金霉素(CTC); (5)林可酰胺类:林可霉素(LCM); (6)大环内酯类:罗红霉素(ROX)、红霉素(ERY)、替米考星(TIL)、螺旋霉素(SPM)、新螺旋霉素(NSPM); (7)硝基咪唑类:地美硝唑(DMZ)、洛硝达唑(RNZ)、甲硝唑(MNZ)、羟基甲硝唑(MNZOH); (8)双萜烯类:泰妙菌素(TML)、沃尼妙林(VLM); (9)二氢叶酸还原酶抑制剂:甲氧苄啶(TMP)。以上标准品均购自北京坛墨科技有限公司,四环素类为固体粉末,纯度大于95%,其余均为标准溶液,质量浓度为100 μg/mL。

12种内标:磺胺甲恶唑-^13^C_6_(SMX-^13^C_6_)、磺胺二甲基嘧啶-^13^C_6_(SMZ-^13^C_6_)、磺胺喹噁啉-^13^C_6_(SQ-^13^C_6_)、磺胺间甲氧嘧啶-^13^C_6_(SMM-^13^C_6_)、磺胺间二甲氧嘧啶-D_6_(SDM-D_6_)。以上标准品均购自天津阿尔塔科技有限公司,质量浓度为100 μg/mL。地美硝唑-D_3_(DMZ-D_3_)、甲硝唑-D_4_(MNZ-D_4_)、磺胺邻二甲氧嘧啶-D_3_(SDX-D_3_)、诺氟沙星-D_5_(NOR-D_5_)、环丙沙星-D_8_(CIP-D_8_)、恩诺沙星-D_5_(ENR-D_5_)、泰妙菌素-^13^C_4_(TML-^13^C_4_)。以上标准品纯度均大于95%,购自北京坛墨科技有限公司。

购买的标准溶液直接使用;粉末标准品则用甲醇配制成1000 mg/L的标准储备液,储备液于-20 ℃冰箱保存,使用时用甲醇稀释至所需浓度。内标混合工作液由甲醇配制而成,标准系列工作溶液则采用含0.1%甲酸的甲醇-水(5∶95, v/v)混合溶液配制而成。

### 1.2 样品前处理

水样经0.45 μm水相滤膜过滤,量取水样0.5 L,加入50 μL 100 μg/L的内标混合工作液,再分别加入2.92 g磷酸二氢钠和0.25 g Na_2_EDTA充分混匀,用磷酸调节pH至2.34。依次用10 mL甲醇、10 mL纯水活化Oasis HLB固相萃取柱,通过自动上样装置,以5~8 mL/min的流速上样,上样完毕后,使用10 mL纯水淋洗,在负压下干燥小柱20 min后,用10 mL甲醇进行洗脱,并收集全部的洗脱液于15 mL离心管中,氮气吹至近干。加入1 mL含0.1%甲酸的甲醇-水(5∶95, v/v)混合溶液溶解,超声混匀后,经0.22 μm滤膜过滤,供超高效液相色谱-串联质谱仪分析测定。

### 1.3 分析条件

#### 1.3.1 色谱条件

色谱柱: Acquity UPLC BEH C_18_色谱柱(50 mm×2.1 mm, 1.7 μm);流动相:A为0.1%甲酸水溶液,B为含0.1%甲酸的甲醇-乙腈(2∶8, v/v)混合溶液;梯度洗脱程序:0~2.0 min, 5%B; 2.0~5.0 min, 5%B~15%B; 5.0~7.0 min, 15%B~40%B; 7.0~7.1 min, 40%B~5%B; 7.1~9.0 min, 5%B。柱温:40 ℃;流速:0.3 mL/min;进样量:10 μL。

#### 1.3.2 质谱条件

采用电喷雾电离源(ESI),正离子扫描,脱溶剂温度为500 ℃,毛细管电压为3 kV,脱溶剂气流量800 L/h,锥孔气流速150 L/h,扫描方式为多反应监测模式(MRM),手动分段扫描。各化合物的质谱参数见表1。

**表1:** 43种抗菌药物及内标的质谱参数

Compound	*t*_R_/min	Parent ion (*m/z*)	Daughter ion (*m/z*)	Cone voltage/V	Collision energy/V
Sulfonamides (SAs)					
Sulfamethoxazole (SMX)	3.48	254.1	92.0	30	25
			156.2^*^	30	15
Sulfamethazine (SMZ)	2.62	279.1	124.1	35	25
			186.0^*^	35	15
Sulfamonomethoxine (SMM)	3.17	281.0	92.0	35	35
			156.2^*^	35	22
Sulfaquinoxaline (SQ)	4.39	301.1	92.2	32	30
			156.1^*^	32	16
Sulfadimethoxine (SDM)	4.33	311.1	92.0	36	32
			156.0^*^	36	20
Sulfadiazine (SDZ)	1.53	251.0	92.0	30	27
			156.0^*^	30	15
Sulfathiazole (STZ)	1.81	256.1	92.0	30	30
			156.0^*^	30	18
Sulfamerazine (SMR)	2.08	265.1	92.0	35	25
			156.0^*^	35	15
Sulfisoxazole (SIZ)	3.75	268.1	92.0	30	34
			156.0^*^	30	18
Sulfamethizole (SMTZ)	2.66	271.0	92.0	20	35
			156.0^*^	20	18
Sulfadoxine (SDX)	3.53	311.0	92.0	30	32
			156.0^*^	30	15
Sulfachloropyridazine (SCP)	3.21	285.1	92.0	30	28
			156.0^*^	30	15
Quinolones (QNs)					
Oxolinic acid (OXO)	4.16	262.0	216.0	40	30
			244.0^*^	40	17
Flumequine (FLU)	5.30	262.1	202.0	30	32
			244.0^*^	30	21
Fluoroquinolones (FQs)					
Enrofloxacin (ENR)	3.31	360.2	245.0	32	20
			316.1^*^	32	22
Ciprofloxacin (CIP)	2.84	332.1	231.1	32	45
			314.1^*^	32	22
Ofloxacin (OFL)	2.73	362.1	261.2	42	25
			318.2^*^	42	17
Norfloxacin (NOR)	2.74	320.2	233.1	42	28
			302.2^*^	42	18
Enoxacin (ENO)	2.43	321.0	234.0	37	22
			257.1^*^	37	17
Pefloxacin (PEF)	2.63	334.1	316.2	42	24
			290.2^*^	42	16
Lomefloxacin (LOM)	2.89	352.2	308.2	42	16
			265.2^*^	42	21
Danofloxacin (DAN)	2.89	358.2	314.0	32	20
			96.0^*^	32	25
Flerofloxacin (FLE)	2.52	370.1	269.1	34	25
			326.1^*^	34	19
Sarafloxacin (SAR)	3.33	386.2	299.1	37	27
			342.1^*^	37	18
Sparfloxacin (SPX)	3.45	393.2	292.1	37	24
			349.1^*^	37	20
Difloxacin (DIF)	3.35	400.2	299.0	37	27
			356.1^*^	37	21
Tetracyclines (TCs)					
Doxycycline (DC)	4.12	445.1	410.1	30	24
			154.0^*^	30	25
Tetracycline (TC)	2.86	445.2	427.2	40	15
			410.1^*^	40	19
Oxytetracycline (OTC)	2.62	461.2	443.2	30	14
			426.1^*^	30	18
Chlortetracycline (CTC)	3.80	479.2	154.0	40	25
			444.1^*^	40	20
Lincosamides (LINs)					
Lincomycin (LCM)	2.11	407.3	359.3	45	17
			126.2^*^	45	30
Macrolides (MLs)					
Erythromycin (ERY)	5.15	734.5	116.1	35	32
			158.0^*^	35	46
Roxithromy (ROX)	5.92	837.6	116.1	20	45
			158.2^*^	20	35
Tilmicisin (TIL)	4.64	869.6	132.1	45	45
			174.1^*^	45	42
Spiramycin (SPM)	3.97	843.7	142.1	40	30
			174.2^*^	40	35
Neospiramycin (NSPM)	3.64	699.6	142.2	65	23
			174.2^*^	65	27
Nitroimidazoles (NMs)					
Dimetridazole (DMZ)	1.46	142.0	81.1	30	22
			96.0^*^	30	15
Ronidazole (RNZ)	1.50	201.0	55.0	30	20
			140.0^*^	30	10
Metronidazole (MNZ)	1.25	172.0	82.0	30	21
			128.0^*^	30	15
Hydroxymetronidazole (MNZOH)	1.00	188.1	122.9	30	10
			125.9^*^	30	15
Diterpenes (DTs)					
Tiamulin (TML)	5.55	494.5	119.0	18	38
			192.1^*^	18	18
Valnemulin (VLM)	5.95	565.5	163.9	20	30
			263.2^*^	20	15
Dihydrofolate reductase inhibitors (DRIs)															
Trimethoprim (TMP)	2.38	291.2	261.0	42	23
			230.0^*^	42	21
ISs					
Dimetridazole-D_3_ (DMZ-D_3_)	1.46	145.0	99.0	34	14
Metronidazole-D_4_ (MNZ-D_4_)	1.25	176.1	128.0	25	12
Sulfamethoxazole-^13^C_6_ (SMX-^13^C_6_)	3.48	260.1	162.0	30	15
Sulfamethazine-^13^C_6_ (SMZ-^13^C_6_)	2.62	285.2	186.0	44	16
Sulfamonomethoxine-^13^C_6_ (SMM-^13^C_6_)	3.17	287.1	162.0	2	16
Sulfaquinoxaline-^13^C_6_ (SQ-^13^C_6_)	4.39	307.1	162.0	34	14
Sulfadoxine-D_3_ (SDX-D_3_)	3.53	314.0	156.0	45	24
Sulfadimethoxine-D_6_ (SDM-D_6_)	4.33	317.2	162.1	6	20
Norfloxacin-D_5_ (NOR-D_5_)	2.56	325.1	281.1	30	18
Ciprofloxacin-D_8_ (CIP-D_8_)	2.69	340.2	249.1	46	24
Enrofloxacin-D_5_ (ENR-D_5_)	3.00	365.2	245.2	16	28
Tiamulin-^13^C_4_ (TML-^13^C_4_)	5.55	498.5	196.1	36	20

* Quantitative ion.

## 2 结果与讨论

### 2.1 仪器条件优化

配制一定浓度的标准溶液,根据各化合物及内标的分子结构特征,在正离子扫描模式下通过流动注射进入质谱进行扫描,确定最佳的毛细管电压、锥孔电压、脱溶剂温度、母离子、子离子、碰撞能力等质谱参数(见表1)。

对液相色谱分离条件进行优化,有机相采用纯乙腈分离效果不如甲醇-乙腈(2∶8, v/v)混合溶液。加入甲酸可以使大多数碱性化合物质谱电离效果更好,最终在水相、有机相中分别添加0.1%的甲酸。为了保证每种物质都有足够的驻留时间,质谱采集时间确定为9 min,并对采集时间进行手动分段,自动分配驻留时间模式下,各物质的驻留时间在0.005~0.027 s之间,色谱峰采集点数为12.16,满足定性、定量要求。43种抗菌药物的总离子流色谱图见图1。

**图1:**
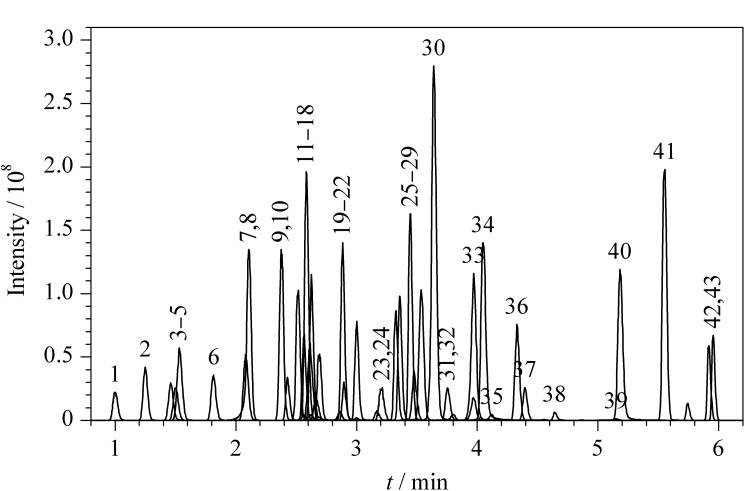
43种抗菌药物(5 μg/L)的总离子流色谱图

### 2.2 自动上样装置的设计

检测水中抗菌药物时,需要用固相萃取柱对目标物进行富集、净化。由于上样体积比较大,整个上样过程操作麻烦、费时费力且不可控。为了解决该难题,本文设计了一套自动上样装置。该自动上样装置主要由蓄水瓶、导流软管、液体缓冲区、流速调节器、适配器等部件组成。流速调节器主要控制上样速度,上样速度可通过目视液体缓冲区液滴速度来判断(具体流速可使用秒表、量筒进行校准)。导流软管下端密闭连接适配器,可实现与不同规格的固相萃取柱的密闭连接。具体设计方案见图2。该自制的自动上样装置与固相萃取装置相耦合,从而实现大体积水样前处理过程的自动化。本装置具有操作方便、稳定性好且成本低等特点,具有很强的实用性,可推广、可复制,适用于大体积样品的固相萃取过程,有助于提高萃取效率,以及保证实验结果的有效性。

**图2:**
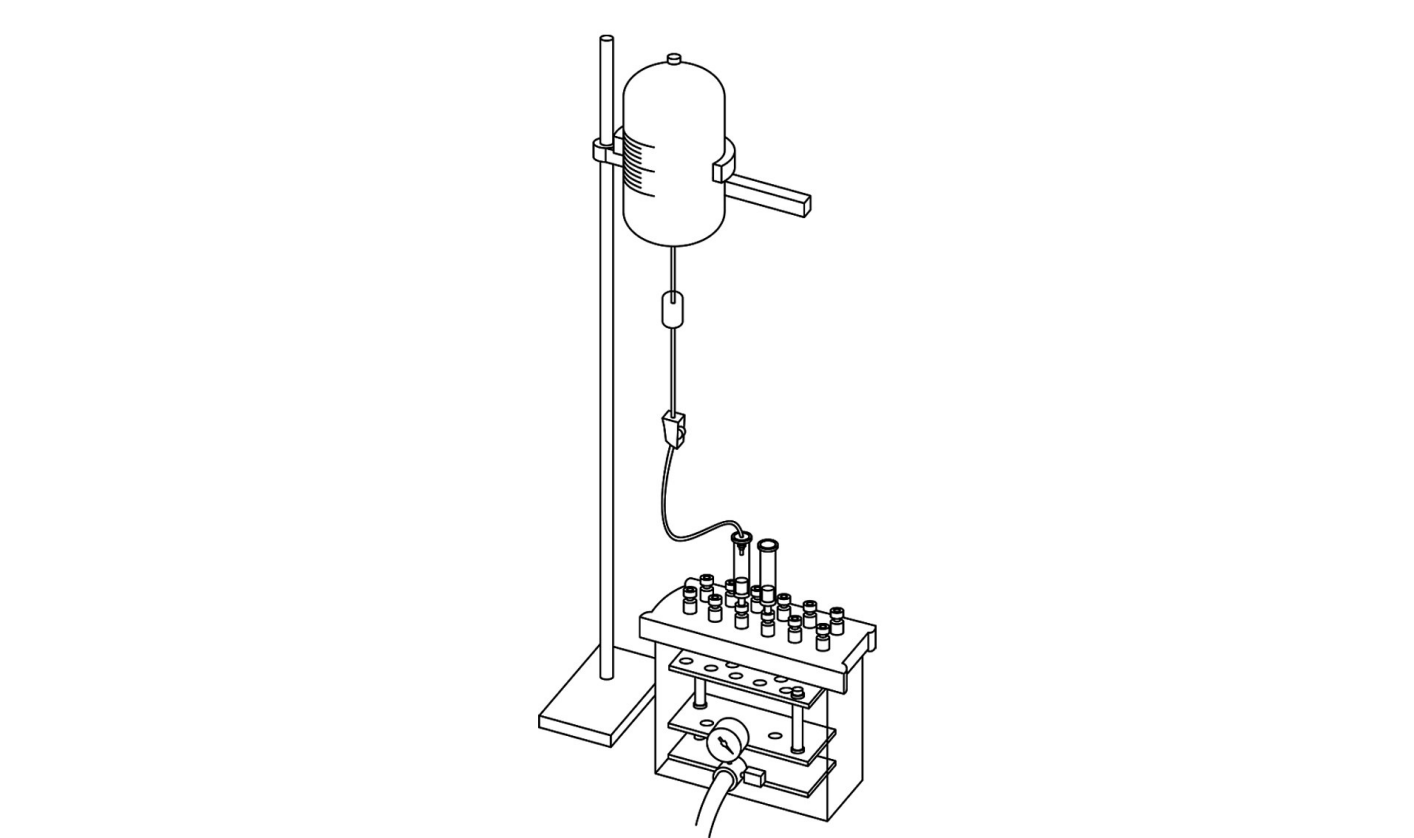
自动上样装置示意图

### 2.3 前处理条件的优化

四环素类、氟喹诺酮类、大环内酯类抗生素容易与金属离子螯合,影响回收率,本实验选择Na_2_EDTA作为螯合剂。磷酸二氢钠具有一定的缓冲效果,可以保证水中pH值的稳定。43种抗菌药物性质差异较大,为了实现水中抗菌药物的高通量检测,本实验对固相萃取柱类型、pH值、上样量等参数进行了优化。为了避免基质效应,选用去离子水作为基质进行加标试验。除了不添加内标外,按照1.2节进行样品前处理,以回收率作为评价指标,确定较合理的前处理条件。

#### 2.3.1 固相萃取柱及水样pH值的选择

Oasis HLB柱填料为二乙烯基苯-乙烯基吡咯烷酮,是一种亲水、亲酯性大孔聚合填料,具有耐酸碱特性,通常在pH 1~14范围内都能保持稳定,常被用作多残留药物的富集净化。Oasis MCX柱以磺酸化的聚苯乙烯-二乙烯基苯为填料,是一种混合型固相萃取填料,具有非极性及阳离子交换功能。两者的洗脱溶剂不同,其中Oasis HLB柱选择10 mL甲醇进行洗脱,而Oasis MCX柱则分别用5 mL纯甲醇和5 mL 5%氨水甲醇依次洗脱。

对于Oasis HLB柱,本文考察了pH值为2.34、4.60(未进行酸碱调整时的pH值)、7.00、9.00时各物质的回收率。环境体系的pH比目标化合物的p*K*_a_至少小于2才能保证弱碱性化合物99%解离为阳离子^[11]^,因此,对于Oasis MCX柱,只考虑pH值为2.34条件下各化合物的回收率。本文涉及的物质多达43种,考虑到每类物质性质相近,受pH值、SPE柱类型的影响亦相近,因此从每类化合物中选取代表性抗菌药物,考察其受pH值、SPE柱类型等因素的影响规律。pH值及SPE柱对去离子水基质中部分抗菌药物回收率的影响见图3。结果显示,磺胺类与喹诺酮类在所有条件下都保持较高的回收率。氟喹诺酮类在其母核喹啉上有一个呈酸性的羧基和一个呈碱性的哌嗪环,是一类两性化合物,p*K*_a_值分别为5.5~6.6和7.2~10.2^[12]^。当pH值为2.34时,氟喹诺酮类以阳离子形式存在,可以以非共价键及离子作用力与Oasis MCX结合,得到较高的回收率。使用Oasis HLB柱时,氟喹诺酮类的回收率随着pH值升高存在降低的趋势。

**图3:**
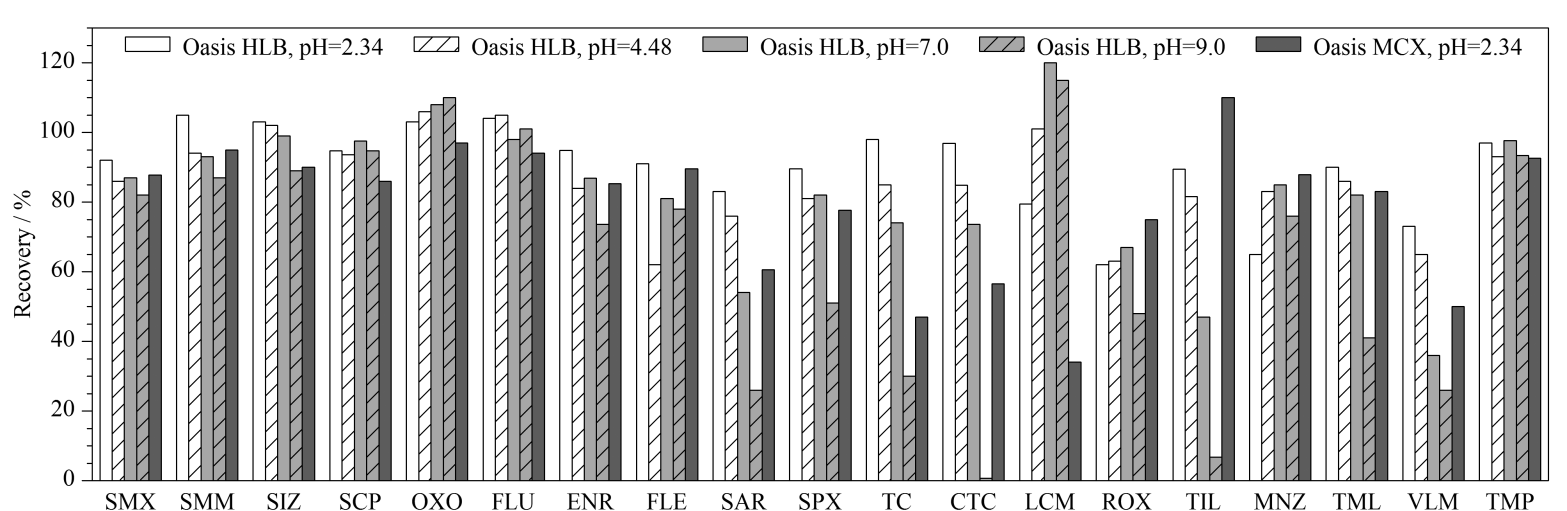
pH值及固相萃取柱对代表性抗菌药物回收率的影响

对于四环素类,使用Oasis HLB柱,当pH值为2.34时,回收率最高;随着pH值升高,回收率下降。特别是pH达到9时,回收率非常不理想,主要是碱性环境下四环素类不稳定,容易内酯化^[12]^。使用MCX柱时,4种四环素类物质的回收率均不够理想,可能是使用5%氨水甲醇洗脱的缘故。对于大环内酯类、林可酰胺类、硝基咪唑类化合物,随着pH值的增加,使用Oasis HLB柱的回收率均有所提高,可能是由于它们是弱碱性化合物^[13⇓-15]^,随着pH值的增大,非极性变强,目标物在Oasis HLB柱上的吸附能力增强。pH值达到9时,大环内酯类化合物回收率不够理想,主要是因为它们在碱性条件下会开环降解^[16]^;在酸性条件下,它们均以离子态存在,除林可霉素外,其余化合物在MCX柱上也有较好的吸附。双萜烯类化合物也具有一定弱碱性^[17]^,但是使用HLB柱富集净化的回收率随着pH值的增加反而降低。使用MCX柱富集净化时,双萜烯类的回收率较为理想。

综合以上分析,为了实现43种抗菌药物的同步检测,选用Oasis HLB柱进行目标物富集净化,pH值不宜超过4.48。考虑到四环素、双萜烯类、氟喹诺酮类等化合物在低pH值下有更高的回收率,最终确定水样pH值为2.34左右。

#### 2.3.2 上样体积的考察

SPE方法富集的水样体积主要受限于SPE柱的类型以及柱容量。本实验选用Oasis HLB柱,在pH值为2.34情况下,比较了上样量对回收率的影响。上样量分别为0.5、1.0、1.5 L时,考察各物质的回收率,从而确定最佳的上样体积。结果发现,林可霉素、羟基甲硝唑、甲硝唑、地美硝唑受上样体积影响较大。上样体积为1.5 L时,林可霉素的回收率为46.6%。上样体积为1.0 L时,羟基甲硝唑、甲硝唑、地美硝唑三者的绝对回收率分别为30.9%、39.5%、55.4%,主要是因为三者具有较强极性。因此,综合考虑方法灵敏度、回收率等因素,本研究最终确定上样体积为0.5 L。

### 2.4 方法学验证

#### 2.4.1 标准曲线和检出限

以加标回收率为评价标准,为每种化合物选择合适的内标物。因多西环素、西环素、土霉素、金霉素、替米考星等5种化合物的内标法定量结果(匹配任何一种内标物)准确性均不如外标法,因此该5种化合物采用外标法定量,其余化合物均采用内标法定量,各化合物内标物匹配情况见表2。根据各物质的灵敏度,配制一定梯度的系列混合标准溶液,上机测试。内标法以目标物峰面积与内标物峰面积比值为纵坐标(*y*),外标法以目标物峰面积为纵坐标(*Y*),均以质量浓度为横坐标(*x*, μg/L)绘制标准曲线。以信噪比大于3定义为方法的检出限(LOD),信噪比大于10定义为方法的定量限(LOQ)。结果表明,所有物质在各自的线性范围内线性均较好,线性相关系数(*r*^2^)为0.9965~0.9999,43种抗菌药物的LOD为0.004~1.000 ng/L,LOQ为0.012~3.000 ng/L,结果见表2。

**表2:** 43种抗菌药物的线性范围、回归方程、相关系数、检出限和定量限

Compound	Linear range/(μg/L)	Regression equation	*r*^2^	LOD/(ng/L)	LOQ/(ng/L)	IS
SMX	0.01-50	*y*=0.94601*x*+0.0023	0.9993	0.020	0.060	SMX-^13^C_6_
SMZ	0.05-50	*y*=1.01231*x*+0.0015	0.9997	0.100	0.300	SMZ-^13^C_6_
SMM	0.5-50	*y*=0.26836*x*+0.0003	0.9995	1.000	3.000	SMM-^13^C_6_
SQ	0.05-50	*y*=1.01851*x*+0.0085	0.9997	0.100	0.300	SQ-^13^C_6_
SDM	0.005-50	*y*=1.85311*x*+0.0083	0.9986	0.010	0.030	SDM-D_6_
SDZ	0.005-50	*y*=1.51077*x*+0.0060	0.9981	0.010	0.030	SMX-^13^C_6_
STZ	0.01-50	*y*=0.48650*x*+0.0004	0.9999	0.020	0.060	SMZ-^13^C_6_
SMR	0.005-50	*y*=0.68671*x*+0.0004	0.9986	0.010	0.030	SMZ-13C_6_
SIZ	0.01-20	*y*=0.79491*x*+0.0021	0.9966	0.020	0.060	SMM-^13^C_6_
SMTZ	0.05-50	*y*=0.31235*x*+0.0007	0.9999	0.100	0.300	SMZ-^13^C_6_
SDX	0.01-50	*y*=2.97870*x*+0.0829	0.9965	0.020	0.060	SDX-D_3_
SCP	0.01-50	*y*=1.03829*x*+0.0020	0.9994	0.020	0.060	SMM-^13^C_6_
OXO	0.01-10	*y*=4.85260*x*-0.0040	0.9997	0.020	0.060	ENR-D_5_
FLU	0.01-10	*y*=2.98750*x*-0.0024	0.9997	0.020	0.060	ENR-D_5_
ENR	0.01-10	*y*=1.48110*x*+0.0127	0.9997	0.020	0.060	ENR-D_5_
CIP	0.01-10	*y*=1.48110*x*+0.0127	0.9997	0.020	0.060	CIP-D_8_
OFL	0.01-10	*y*=3.23850*x*+0.0032	0.9995	0.020	0.060	ENR-D_5_
NOR	0.05-20	*y*=1.29990*x*-0.0062	0.9994	0.100	0.300	NOR- D_5_
ENO	0.01-10	*y*=0.48160*x*+0.0012	0.9992	0.020	0.060	ENR-D_5_
PEF	0.01-10	*y*=1.70030*x*+0.0069	0.9997	0.020	0.060	ENR-D_5_
LOM	0.01-10	*y*=2.23480*x*+0.0152	0.9996	0.020	0.060	ENR-D_5_
DAN	0.01-10	*y*=0.46830*x*+0.0020	0.9997	0.020	0.060	ENR-D_5_
FLE	0.01-10	*y*=1.88390*x*+0.0001	0.9999	0.020	0.060	ENR-D_5_
SAR	0.01-10	*y*=1.35300*x*-0.0018	0.998	0.020	0.060	ENR-D_5_
SPX	0.01-10	*y*=2.56490*x*+0.0038	0.9997	0.020	0.060	ENR-D_5_
DIF	0.01-10	*y*=1.49440*x*-0.0020	0.9996	0.020	0.060	ENR-D_5_
DC	0.5-50	*Y*=34848.5*x*+25.395	0.9997	1.000	3.000	-
TC	0.5-50	*Y*=41592.4*x*-14.051	0.9986	1.000	3.000	-
OTC	0.5-50	*Y*=27170.0*x*-61.586	0.9979	1.000	3.000	-
CTC	0.05-50	*Y*=323489*x*-79.154	0.9996	0.100	0.300	-
LCM	0.01-5.0	*y*=12.8809*x*-79.154	0.9986	0.020	0.060	TIA-^13^C_4_
ERY	0.05-50	*y*=0.09243*x*-0.0001	0.9989	0.100	0.300	TIA-^13^C_4_
ROX	0.01-10	*y*=2.94440*x*-0.0155	0.9995	0.020	0.060	TIA-^13^C_4_
TIL	0.05-20	*Y*=36093.3*x*-586.19	0.9968	0.100	0.300	-
SPM	0.01-10	*y*=3.61288*x*-0.0313	0.9995	0.020	0.060	TIA-^13^C_4_
NSPM	0.01-10	*y*=5.91638*x*-0.0230	0.9989	0.020	0.060	TIA-^13^C_4_
DMZ	0.01-20	*y*=1.73280*x*+0.0448	0.9989	0.020	0.060	DMZ-D_3_
RNZ	0.01-20	*y*=1.38934*x*+0.0101	0.9978	0.020	0.060	DMZ-D_3_
MNZ	0.01-20	*y*=1.75019*x*+0.0048	0.9997	0.020	0.060	MNZ-D_4_
MNZOH	0.01-20	*y*=0.84597*x*-0.0021	0.9999	0.020	0.060	MNZ-D_4_
TLM	0.002-5.0	*y*=13.1517*x*+0.0457	0.9986	0.004	0.012	TIA-^13^C_4_
VLM	0.005-10	*y*=3.87296*x*+0.0006	0.9999	0.010	0.030	TIA-^13^C_4_
TMP	0.01-50	*y*=1.86337*x*+0.0140	0.9975	0.020	0.060	SMZ-^13^C_6_

*Y*: peak area; *y*: peak area ratio of compound to internal standard; *x*: mass concentration, μg/L; -: no IS.

#### 2.4.2 加标回收率

选择长江江阴段、锡澄运河江阴段的水样以及自来水作为基质,进行加标回收试验,加标水平为10 ng/L和20 ng/L,每份样品重复实验3次,考察方法的加标回收率以及精密度,结果见表3。结果表明,各化合物的加标回收率为53.7%~130.4%,相对标准偏差为0.9%~13.2%;其中羟基甲硝唑的回收率最低,主要是由于其极性最强,在SPE柱上不易保留。上述结果表明,该方法对环境水样以及生活饮用水中43种抗菌药物的残留检测均具有较好的准确度和精密度。

**表3:** 3个水样中43种抗菌药物在2个水平下的加标回收率和相对标准偏差(*n*=3)

Compound		Recoveries (RSDs)/%																						
		Yangtze River									Xicheng Canal										Tap water			
	10 ng/L		20 ng/L							10 ng/L					20 ng/L							10 ng/L		20 ng/L	
SMX	109.3 (3.8)		103.4 (2.8)							100.3 (5.9)				97.6 (3.2)								95.4 (2.1)		99.0 (1.8)
SMZ	100.2 (4.5)		100.3 (2.0)							107.2 (4.5)				100.3 (3.2)								110.3 (2.3)		109.4 (4.1)
SMM	97.2 (3.1)		94.2 (3.4)							95.8 (4.9)				93.2 (4.0)								109.7 (2.5)		105.7 (3.7)
SQX	109.2 (5.2)		110.2 (9.3)							115.0 (9.5)				113.2 (8.3)								107.4 (5.2)		99.8 (1.9)
SDM	115.8 (10.2)		117.3 (4.5)							113.2 (5.9)				109.2 (2.5)								109.2 (4.3)		110.2 (7.4)
SDZ	76.3 (6.5)		81.4 (6.9)							83.7 (7.3)				85.9 (3.4)								89.6 (6.8)		90.4 (2.4)
STZ	71.0 (11.2)		73.2 (6.0)							72.4 (3.1)				70.4 (10.3)								79.4 (5.3)		84.3 (7.6)
SMR	94.2 (8.7)		95.7 (4.3)							106.6 (7.4)				102.1 (3.5)								104.5 (8.3)		108.9 (6.9)
SIZ	82.0 (2.0)		84.2 (8.5)							85.2 (9.1)				86.9 (4.5)								84.3 (6.5)		83.2 (4.9)
SMTZ	83.2 (13.2)		82.9 (11.5)							90.5 (3.8)				90.1 (6.3)								97.3 (6.2)		90.9 (9.0)
SDX	109.2 (2.3)		110.2 (4.5)							100.3 (1.1)				109.3 (6.4)								105.1 (5.4)		108.1 (9.6)
SCP	110.7 (11.8)		107.7 (9.0)							120.1 (4.3)				115.9 (7.1)								110.1 (12.4)		109.3 (11.4)
OXO	130.4 (7.3)		127.6 (3.5)							120.3 (6.6)				123.4 (2.6)								110.2 (5.6)		115.3 (4.9)
FLU	127.4 (3.9)		129.5 (7.3)							117.3 (4.5)				125.4 (4.9)								123.2 (5.3)		118.9 (7.1)
ENR	86.3 (3.9)		90.1 (2.6)							85.4 (4.9)				81.3 (5.1)								87.1 (2.5)		89.3 (3.2)
CIP	110.5 (2.3)		117.0 (5.0)							117.6 (6.9)				113.3 (5.1)								110.3 (7.5)		107.6 (8.1)
OFL	119.2 (7.3)		120.3 (7.4)							115.3 (7.2)				105.7 (4.3)								102.1 (8.9)		93.8 (6.8)
NOR	115.3 (9.3)		109.3 (8.4)							116.3 (12.4)				110.2 (6.6)								110.3 (7.5)		115.3 (5.3)
ENO	115.3 (7.6)		107.1 (5.3)							100.2 (3.5)				100.2 (3.5)								107.3 (2.9)		100.2 (3.7)
PEF	100.3 (4.1)		101.1 (3.4)							97.1 (7.1)				102.7 (4.5)								104.3 (5.9)		98.3 (7.0)
LOM	113.4 (4.6)		110.5 (8.2)							100.1 (3.2)				100.2 (7.1)								110.3 (9.5)		107.4 (7.9)
DAN	89.3 (9.3)		87.2 (9.4)							87.9 (6.4)				89.2 (3.5)								83.3 (5.0)		85.3 (7.4)
FLE	97.3 (2.6)		99.3 (4.5)							95.1 (5.2)				95.0 (4.2)								99.3 (3.9)		100.2 (10.7)
SAR	104.9 (5.7)		100.5 (7.3)							97.3 (4.5)				95.1 (6.1)								103.2 (8.0)		109.4 (7.6)
SPX	86.7 (4.0)		90.6 (9.3)							70.5 (5.1)				71.4 (5.3)								88.3 (9.0)		90.5 (6.5)
DIF	95.2 (4.9)		100.2 (2.4)							87.1 (4.0)				85.2 (5.9)								89.2 (4.0)		90.3 (8.6)
DC	79.3 (9.2)		70.3 (8.4)							77.3 (10.3)				78.9 (9.4)								80.3 (13.7)		82.1 (9.8)
TC	124.6 (10.4)		130.4 (9.6)							123.4 (8.1)				127.4 (7.4)								117.3 (9.8)		120.4 (9.6)
OTC	121.3 (12.3)		126.4 (10.4)							125.1 (9.1)				120.3 (12.4)								119.3 (7.0)		112.3 (8.6)
CTC	95.4 (9.9)		94.3 (7.2)							97.2 (6.4)				100.3 (6.4)								99.2 (9.1)		104.1 (8.5)
LCM	108.4 (4.3)		-							119.7 (2.7)				-								112.3 (6.9)		-
ERY	60.3 (7.9)		61.9 (4.5)							89.3 (9.0)				90.1 (5.0)								79.1 (4.3)		77.3 (6.0)
ROX	77.5 (6.5)		75.4 (5.4)							85.7 (3.2)				88.5 (4.7)								79.3 (8.7)		77.0 (5.5)
TIL	123.3 (8.8)		129.0 (11.2)							96.5 (2.3)				110.0 (3.3)								87.6 (8.0)		93.9 (8.4)
SPM	68.3 (4.0)		71.2 (5.9)							127.5 (9.4)				120.3 (4.3)								119.3 (12.8)		110.1 (11.6)
NSPM	79.3 (2.5)		79.4 (7.8)							59.7 (10.4)				61.3 (5.8)								86.4 (7.9)		93.0 (9.5)
DMZ	105.5 (4.9)		110.3 (8.9)							114.1 (4.6)				105.3 (8.3)								86.3 (5.3)		82.7 (2.3)
RNZ	74.2 (3.2)		74.3 (9.0)							75.9 (5.8)				77.8 (5.2)								88.9 (5.1)		93.7 (4.0)
MNZ	89.3 (4.8)		90.1 (9.5)							95.4 (8.0)				89.0 (6.4)								86.8 (1.9)		84.3 (6.1)
MNZOH	59.3 (5.4)		53.7 (3.6)							64.3 (7.3)				58.7 (4.1)								70.3 (5.3)		79.1 (5.2)
TLM	90.3 (1.9)		-							103.0 (3.6)				-								94.3 (7.6)		-
VLM	99.2 (1.5)		99.1 (1.3)							94.2 (1.6)				100.2 (0.9)								93.5 (6.8)		94.9 (4.9)
TMP	77.7 (7.4)		82.2 (5.3)							80.6 (8.7)				79.3 (2.9)								86.1 (2.5)		83.1 (9.5)

-: no data.

### 2.5 实际水样检测

应用本方法对长江江阴段3个点位、锡澄运河江阴段3个点位共计6份水样以及取自不同区域的6份自来水开展43种抗菌药物的监测,结果见表4。6份来自长江水及锡澄运河的水样中共检出了20种抗菌药物。除四环素类,其余类别均有检出,其中,以磺胺甲恶唑的检出值最大,含量为8.92~11.03 ng/L。锡澄运河XC_1_点位实际水样的总离子流色谱图见图4。6份自来水中均未检测到目标物。通过比较,锡澄运河水中检测到的抗菌药物类别、含量均高于长江。值得注意的是,在长江和锡澄运河水体中均检测到了较高含量的泰妙菌素和沃尼妙林。

**表4:** 12份实际水样中抗菌药物的检测结果^*^

Site	Contents/(ng/L)																			
	SMX	SMZ	SMM	SDZ	SDX	SCP	OXO	FLU	ENR	OFL	LCM	ERY	ROX	TIL	DMZ	RNZ	MNZ	TML	VLM	TMP
YR_1_	10.00	0.12	3.22	0.22	0.02	0.60	-	0.10	0.14	0.28	2.64	0.78	0.20	-	1.06	-	0.72	0.10	1.86	0.24
YR_2_	9.32	0.15	2.63	0.23	-	1.32	-	-	0.22	0.52	2.51	0.89	0.32	-	1.30	-	0.81	0.15	1.73	0.34
YR_3_	11.03	0.17	2.81	0.21	-	1.14	-	-	0.19	0.40	2.59	0.76	-	-	1.59	-	0.77	0.20	1.90	0.30
XC_1_	10.15	0.12	3.20	0.22	0.04	0.58	0.16	0.16	-	1.60	3.50	1.20	0.40	0.08	1.46	0.52	-	0.12	8.91	0.23
XC_2_	9.73	0.13	2.53	0.19	-	1.22	0.23	0.36	-	2.19	3.90	1.06	0.45	0.02	1.21	0.61	-	0.15	7.60	0.22
XC_3_	8.92	0.12	1.92	0.18	-	1.51	0.22	0.15	-	1.70	2.90	1.03	0.41	0.08	1.52	0.77	-	0.16	8.06	0.21

* No target analyte was detected in the six tap water samples. YR: Yangtze River; XC: Xicheng Canal; -: not detected.

**图4:**
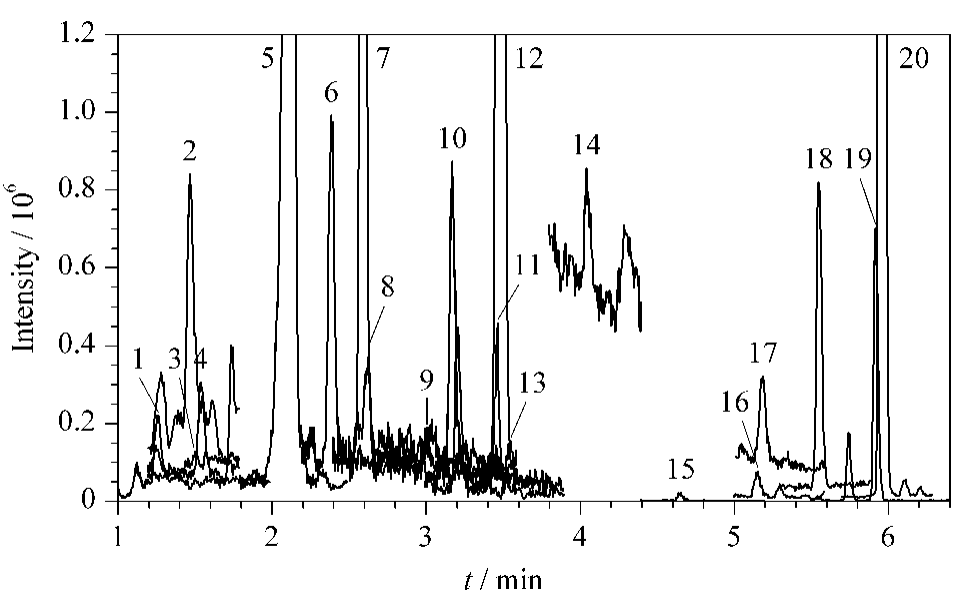
锡澄运河XC_1_点位实际水样的总离子流色谱图

## 3 结论

本文建立了自动上样固相萃取-超高效液相色谱-串联质谱法同时测定水中9类43种抗菌药物残留量的分析方法。该方法操作简单,灵敏度高,稳定性好,适用于多种水体中抗菌药物残留量的检测,为水体中抗菌药物暴露水平的监测提供了技术保障。
